# Genomic Analysis of Mycobacterium abscessus Complex Isolates from Patients with Pulmonary Infection in China

**DOI:** 10.1128/spectrum.00118-22

**Published:** 2022-07-12

**Authors:** Peipei Jin, Jing Dai, Yinjuan Guo, Xuefeng Wang, Jing Lu, Yan Zhu, Fangyou Yu

**Affiliations:** a Department of Laboratory Medicine, Ruijin Hospital, Shanghai Jiao Tong University School of Medicine, Shanghai, China; b Department of Biochemistry and Pharmacology, University of Melbourne, Melbourne, Victoria, Australia; c Immunity and Infection Program, Department of Microbiology, Biomedicine Discovery Institute, Monash University, Melbourne, Victoria, Australia; d Department of Laboratory Medicine, Shanghai Pulmonary Hospital, Tongji University School of Medicine, Shanghai, China; National University Hospital

**Keywords:** *Mycobacterium abscessus* complex, genomics, phylogenetic tree, antimicrobial resistance, virulence, mobile elements

## Abstract

Members of the Mycobacterium abscessus complex (MABC) are multidrug-resistant nontuberculous mycobacteria and increasingly cause opportunistic pulmonary infections. However, the genetic typing of MABC isolates remains largely unclear in China. Genomic analyses were conducted for 69 MABC clinical isolates obtained from patients with lower respiratory tract infections in Shanghai Pulmonary Hospital between 2014 and 2016. The draft genomes of the 69 clinical strains were assembled, with a total length of 4.5 to 5.6 Mb, a percent GC content (GC%) ranging from 63.9 to 68.1%, and 4,492 to 5,404 genes per genome. Susceptibility test shows that most isolates are resistant to many antimicrobials, including clarithromycin, but susceptible to tigecycline. Analyses revealed the presence of genes conferring resistance to antibiotics, including macrolides, aminoglycosides, rifampicin, and tetracyclines. Furthermore, 80 to 114 virulence genes were identified per genome, including those related to the invasion of macrophages, iron incorporation, and avoidance of immune clearance. Mobile genetic elements, including insertion sequences, transposons, and genomic islands, were discovered in the genomes. Phylogenetic analyses of all MABC isolates with another 41 complete MABC genomes identified three clades; 46 isolates were clustered in clade I, corresponding to M. abscessus subsp. *abscessus*, and 25 strains belonged to existing clonal complexes. Overall, this is the first comparative genomic analysis of MABC clinical isolates in China. These results show significant intraspecies variations in genetic determinants encoding antimicrobial resistance, virulence, and mobile elements and controversial subspecies classification using current marker gene combinations. This information will be useful in understanding the evolution, antimicrobial resistance, and pathogenesis of MABC strains and facilitating future vaccine development and drug design.

**IMPORTANCE** Over the past decade, infections by Mycobacterium abscessus complex (MABC) isolates have been increasingly reported worldwide. MABC strains often show a high incidence in cystic fibrosis (CF) patients, whereas in Asia, these strains are frequently recovered from non-CF patients with significant genomic diversity. The present work involves analyses of the antimicrobial resistance, virulence, and phylogeny of 69 selected MABC isolates from non-CF pulmonary patients in Shanghai Pulmonary Hospital by whole-genome sequencing; it represents the first comprehensive investigation of MABC strains in China at the genomic level. These findings highlight the diversity of this group of nontuberculous mycobacteria and provide a mechanistic understanding of evolution and pathogenesis, which is valuable for the development of novel and effective antimicrobial therapies for deadly MABC infections in China.

## INTRODUCTION

The Mycobacterium abscessus complex (MABC) is one of the major nontuberculous mycobacteria (NTM) and has recently gained broad attention due to the growing number of reports of infections worldwide. The MABC exhibits a high level of genomic diversity (1, 2), with three subspecies, M. abscessus subsp. *abscessus*, M. abscessus subsp. *bolletii*, and M. abscessus subsp. *massiliense*, referred to here as M. abscessus, *M. bolletii*, and *M. massiliense*, respectively (3). MABC strains are ubiquitously present in the environment, from water systems and soil to dust (4); recently, MABC strains have shown a rapidly increasing incidence of pulmonary infections, complicated infections of the skin and soft tissues, and disseminated disease with adverse prognoses (5–8). Many virulence factors, including iron acquisition, phospholipase C, surface glycopeptidolipids, and biofilm formation, concomitantly contribute to MABC infections (9). In addition, treatment of MABC infections remains highly challenging due to the resistance of this group of microorganisms to many antimicrobials. Resistance can be mediated by the impermeability of the mycobacterial cell wall, efflux pumps, antibiotic-modifying enzymes, and/or mutations of drug targets (10). The high rates of antimicrobial resistance of MABC strains can render many combination treatments ineffective (11). Therefore, it is critical to elucidate the mechanisms of antimicrobial resistance and virulence to develop novel and effective treatments.

Previous studies have shown that the MABC accounts for 22.5% of all NTM clinical isolates, and the rate of NTM infections among suspected tuberculosis cases achieves 6.3% in China (12, 13). MABC isolates have been frequently isolated from patients with cystic fibrosis (CF) in Europe and America (14–17), whereas CF is extremely rare in Chinese populations, suggesting that MABC infections in China are mostly limited to non-CF patients (18). Therefore, a systematic analysis of MABC genomes is urgently required to characterize non-CF MABC clinical isolates in China.

In the present study, we conducted a comparative genomic study of 69 MABC clinical isolates obtained from patients with pulmonary infections in Shanghai Pulmonary Hospital (SPH), Shanghai, China. We analyzed critical genetic determinants associated with virulence, antimicrobial resistance, and horizontal gene transfer and the phylogeny of these isolates. These findings provide a mechanistic understanding that could be valuable for developing novel and effective antimicrobial therapies to treat MABC infections.

## RESULTS

### Collection of MABC clinical isolates.

Overall, 86 MABC isolates (see Table S1 in the supplemental material) were collected from 86 patients who were admitted to Shanghai Pulmonary Hospital mainly due to pulmonary infections from 2014 to 2016. Altogether, an analysis of the available demographic information for 64 patients (26 male and 38 female patients) shows that their ages ranged from 22 to 84 years (53.3 ± 15.4 years). Overall, 37 (57.8%), 23 (35.9%), and 4 (6.3%) of the strains were isolated from sputum, bronchoalveolar lavage fluid, and puncture fluid (hydrothorax and pus), respectively. Among the 64 patients, 26 were diagnosed with NTM infection, 27 were diagnosed with pulmonary tuberculosis (15 with secondary pulmonary tuberculosis), and 28 were diagnosed with bronchiectasis. Chest scans revealed abnormal lung shadows in 16 patients, lung cavities in 3 patients, and ground-glass nodules in 1 patient. PCR amplification and sequencing of the *hsp65*, *rpoB*, and 16S rRNA genes indicated that 58, 16, and 12 out of a total of 86 strains belonged to M. abscessus, *M. bolletii*, and *M. massiliense*, respectively.

### Genome sequencing and assembly.

Whole-genome sequencing was conducted for the 86 MABC strains with a depth of 61× to 246× per sample. Five of the samples (UM190121T0113 to -117) were discarded due to insufficient sequencing depths. Eight genomes (UM190527T0031, -32, -58, -59, -62, and -65 and UM190517T0116 and -117) showed extraordinarily large genomes of >6 Mb, suggesting contamination of these DNA samples. Decontamination was conducted (see Materials and Methods); however, subsequent *de novo* assembly using the “clean” reads resulted in severely fragmented genomes except for UM190527T0065. Therefore, only the UM190527T0065 assembly was added back for further analyses. Five genomes (UM190517T0101, -106, -111, -112, and -122) showed <80% average nucleotide identity (ANI) with the reference stain M. abscessus ATCC 19977, indicating that they might be strains other than MABC. Indeed, based on PubMLST predictions, UM190517T0101, -106, -111, -112, and -122 belong to M. intracellulare; they were thus removed from the comparative genomic analysis. Overall, among the 69 remaining isolates, 46, 14, and 9 belong to M. abscessus, *M. bolletii*, and *M. massiliense*, respectively; 16, 6, and 5 sequence types (STs) were identified for each subspecies using PubMLST (Table S2). The resulting 69 genomes have a size of 4.5 to 5.4 Mb, with a percent GC content (GC%) ranging from 63.9 to 68.1% (Table S3). Clusters of Orthologous Genes (COG) analysis shows that these genomes have abundant genes in transcription (347 ± 10), lipid transport and metabolism (344 ± 13), amino acid metabolism (286 ± 8), and secondary metabolism (268 ± 12) (Table S4).

### Antimicrobial resistance.

MIC testing shows that all the 69 isolates are resistant to amoxicillin-clavulanic acid (AMC) and cefepime (FEP), while >80% of the isolates are resistant to ceftriaxone, ciprofloxacin (CIP), imipenem, trimethoprim-sulfamethoxazole (SXT), linezolid (LZD), moxifloxacin (MXF), tobramycin (TOB), doxycycline (DOX), minocycline (MIN), and clarithromycin (Table 1). All the isolates are not resistant to amikacin (AMK), except for UM190527T0037 and UM190527T0042, while 44 have an amikacin MIC of ≤16 mg/L. Approximately 45% of isolates are resistant to cefoxitin (FOX), and only 3 are susceptible (MIC ≤ 16 mg/L). All the strains are susceptible to tigecycline (TGC), with only one exception (UM190527T0042).

**TABLE 1:** Drug susceptibilities of 69 Mycobacterium abscessus complex isolates

Antimicrobial agent	No. (%) of isolates with drug susceptibility			MIC (μg/mL)		
	S^*a*^	I^*a*^	R^*a*^	S	I	R
Imipenem^*b*^		3 (4.41)	65 (95.59)	≤4	8–16	≥32
Linezolid^*b*^	5 (7.35)	2 (2.94)	61 (89.71)	≤8	16	≥32
SXT^*b*^	1 (1.47)		67 (98.53)	≤2/38		≥4/76
Cefoxitin^*b*^	3 (4.41)	34 (50.00)	31 (45.59)	≤16	32–64	≥128
Tobramycin^*b*^		6 (8.82)	62 (91.18)	≤2	4	≥8
Doxycycline^*b*^	3 (4.41)	3 (4.41)	62 (91.18)	≤1	2–4	≥8
Amikacin^*b*^	44 (64.71)	22 (32.35)	2 (2.94)	≤16	32	≥64
Ciprofloxacin^*b*^		2 (2.94)	66 (97.06)	≤1	2	≥4
Moxifloxacin^*b*^	1 (1.47)		67 (98.53)	≤1	2	≥4
Clarithromycin^*b*^	13 (19.12)		55 (80.88)	≤2	4	≥8
AMC^*c*^			68 (100)	≤16	32	≥64
Cefepime^*c*^			68 (100)	≤8	16	≥32
Ceftriaxone^*c*^		1 (1.47)	67 (98.53)	≤16	32	≥64
Minocycline^*c*^	1 (1.47)	3 (4.41)	64 (94.12)	≤1	2–4	≥8
Tigecycline^*c*^	67 (98.53)		1 (1.47)	≤4		>4

a S, susceptible; I, intermediate; R, resistant.

b First-line antimicrobials against MABC isolates.

c Secondary antimicrobials against MABC isolates. Previously reported interpretation criteria are used in this study (49).

Macrolides are recommended to treat NTM infections (19). Interrogating CARD (Comprehensive Antibiotic Resistance Database) with the “strict” algorithm discovered the presence of *erm*(41) gene in 45 genomes, which chromosomally encodes a 23S rRNA methylase and is responsible for inducible clarithromycin resistance. Previous studies identified a T/C polymorphism at nucleotide position 28 of *erm*(41) (20, 21). Among the 45 *erm*(41) genes, 35 belong to the T28 sequevar, while the other 10 are of the C28 sequevar. Strains of the T28 sequevar show a frequency of clarithromycin resistance (90%; 9/10) similar to that of strains of the C28 sequevar (88.5%; 31/35). Most *M. massiliense* strains contain a truncated *erm*(41) gene, as previously reported (22), whereas strain UM190527T0030 does not contain any fragment deletion, yet it still belongs to the C28 sequevar and is susceptible to clarithromycin. Mapping the sequencing reads to the 23S rRNA gene *rrl* (MAB_r5052) identified a number of single nucleotide polymorphisms (SNPs), including an A>G mutation at nucleotide 2271 (nucleotide 2058 in Escherichia coli numbering) in UM190527T0047 and UM190527T0048 and a T>C mutation at nucleotide 2823 (nucleotide 2611 in E. coli numbering) (23) in UM190121T0117, which potentially mediated resistance to macrolide antibiotics, whereas no known resistance-conferring mutations were identified in the 16S rRNA gene *rrs* (MAB_r5051). However, neither the absence/presence of *erm*(41) nor the genetic variations in *rrl* correlate well with the macrolide susceptibility profiles of these strains, suggesting complicated yet unknown mechanisms of antimicrobial resistance in these MABC isolates. With further manual searching, we have discovered that all 69 strains contain *whiB7*, encoding a transcriptional factor that specifically induces the expression of resistance upon treatment with ribosome-targeting antibiotics (24). All strains contain an ADP-ribosyltransferase gene *arr*, conferring resistance to rifampicin. The *aac(2′)* (2′-*N*-acetyltransferase), *eis1* (*N*-acetyltransferase), *eis2* (*N*-acetyltransferase), and *aph(3″)* (3″-phosphotransferase) genes are present in all isolates, potentially conferring resistance to aminoglycosides. All genomes contain *tetX* (flavin adenine dinucleotide [FAD]-binding monooxygenase), conferring resistance to tetracycline, except for UM190527T0030 and UM190527T0051. Arabinosyl transferase genes (*embA*, *embB*, and *embC*) are present in all of the genomes; the genetic variations I303Q and L304M are present in the conserved *embB* resistance-determining region (ERDR) across all genomes, likely conferring resistance to ethambutol.

### Genes encoding virulence.

**FIG 1:**
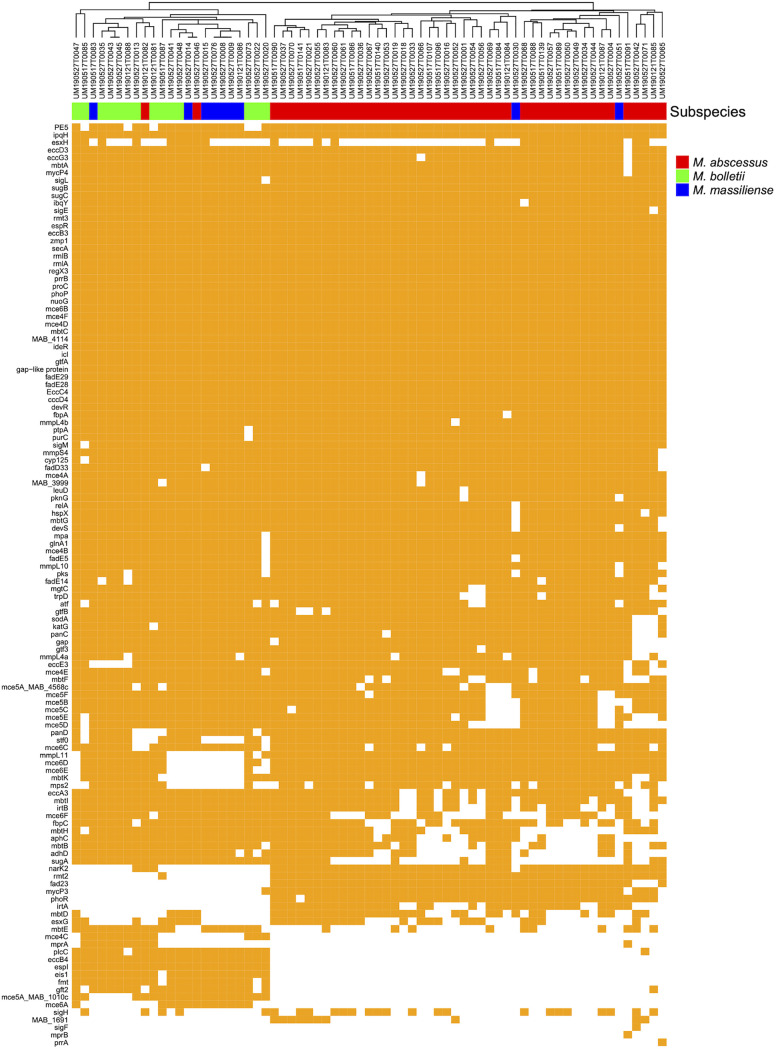
Predicted genetic determinants of virulence in the 69 MABC clinical isolates. Strains were clustered according to the pattern of absence/presence variation.

In total, 80 to 114 virulence-associated genes were identified in the 69 MABC genomes (Fig. 1 and Table S5), encoding various factors required for colonization in host, including adherence, cellular incorporation, and avoidance of immune clearance. Glycopeptidolipid (GPL) is a critical component in the mycomembrane and essential for MABC pathological aspects, including sliding motility, biofilm formation, attachment to host cells, and intracellular trafficking in macrophages (25). Most *M. bolletii* and *M. massiliense* genomes encode a unique *S*-adenosylmethionine (SAM)-dependent methyltransferase (*fmt*) and a glycosyltransferase (*gtf2*), whereas genes for fatty acyl-CoA ligase (*fad23*), peptide synthetase (*mps2*), and SAM-dependent methyltransferase (*rmt2*) are exclusively present in most M. abscessus genomes. The *mmpL11* gene, encoding a cross-membrane heme transporter, is absent in 67% (6/9) of the *M. massiliense* genomes, suggesting alternative heme uptake in this subspecies. Furthermore, the analysis shows that genes for mycobactin biosynthesis are missing in several strains of the three subspecies, particularly those encoding core enzymes, such as *mbtB*, *mbtD*, and *mbtE*. Mycobactin is a critical siderophore produced by mycobacteria for scavenging non-heme iron; the incomplete mycobactin biosynthesis pathway in some MABC strains might indicate the utilization of other siderophores for iron acquisition.

MCE (mammalian cell entry) genes are vital for mycobacterial invasion of host cells as they encode ABC transporters that reside in the extracellular membrane to take up nutrients; genomic analysis revealed the common presence of three *mce* operons in M. abscessus, namely, *mce4 to mce6*; interestingly, *mce4C* is present mostly in *M. bolletii*, while *mce5A* (homolog of MAB_1010c) and *mce6A* selectively exist in non-*abscessus* subspecies. ESX secretion systems are arguably the most studied virulence factors in mycobacteria. Most *esx* genes harbored by these MABC genomes belong to ESX-3 and ESX-4. ESX-4 systems are considered as the ancestral ESX systems from which all of the other systems evolved by gene duplication and diversification (26). Previous studies have indicated that ESX-3 is required for siderophore-mediated iron acquisition, and ESX-4 is critical for the disruption of phagosomes in macrophages (27, 28). Indeed, these two classes are dominant in the 69 MABC strains, with the presence of ESX-1 genes in some of the strains (e.g., *espI* in non-*abscessus* strains) (Fig. 1).

### Mobile genetic elements.

Our analyses predicted 1 to 17 clustered regularly interspaced short palindromic repeat (CRISPR) sequences in 53 of 69 genomes; 27 and 11 genomes contain only 1 and 2 CRISPR sequences, respectively. Interestingly, strains UM190527T0051 and UM190527T0030 possess 10 and 17 CRISPR sequences, respectively, indicating that they might have been exposed to multiple phage infections in their evolutionary history. Overall, 363 insertion sequences (ISs) were predicted for the 69 genomes, including those from the families IS*21* (93 ISs), IS*701* (69), IS*3* (59), and IS*481* (54) (Table S6). A Tn*402*/Tn*5053*-like composite transposon was identified in 4 genomes, UM190517T0084, UM190517T0037, UM190517T0055, and UM190517T0069, which shows high homology (identity of 87.5% and coverage of 52.4%) with part of the Tn*402*-type class 1 integron identified in Proteus mirabilis (Table S7) (29). Furthermore, 713 genomic islands (10.5 ± 3.2 per genome) were identified for 69 genomes (UM190527T0030 showed no genomic island), with an average length ranging from 6,868 to 21,456 bp and a total length per genome ranging from 31,380 to 260,655 bp. Strains UM190527T0022 and UM190527T0051 contain 4 genomic islands, whereas UM190527T0008 contains 17 (Table S8).

### Phylogeny.

**FIG 2:**
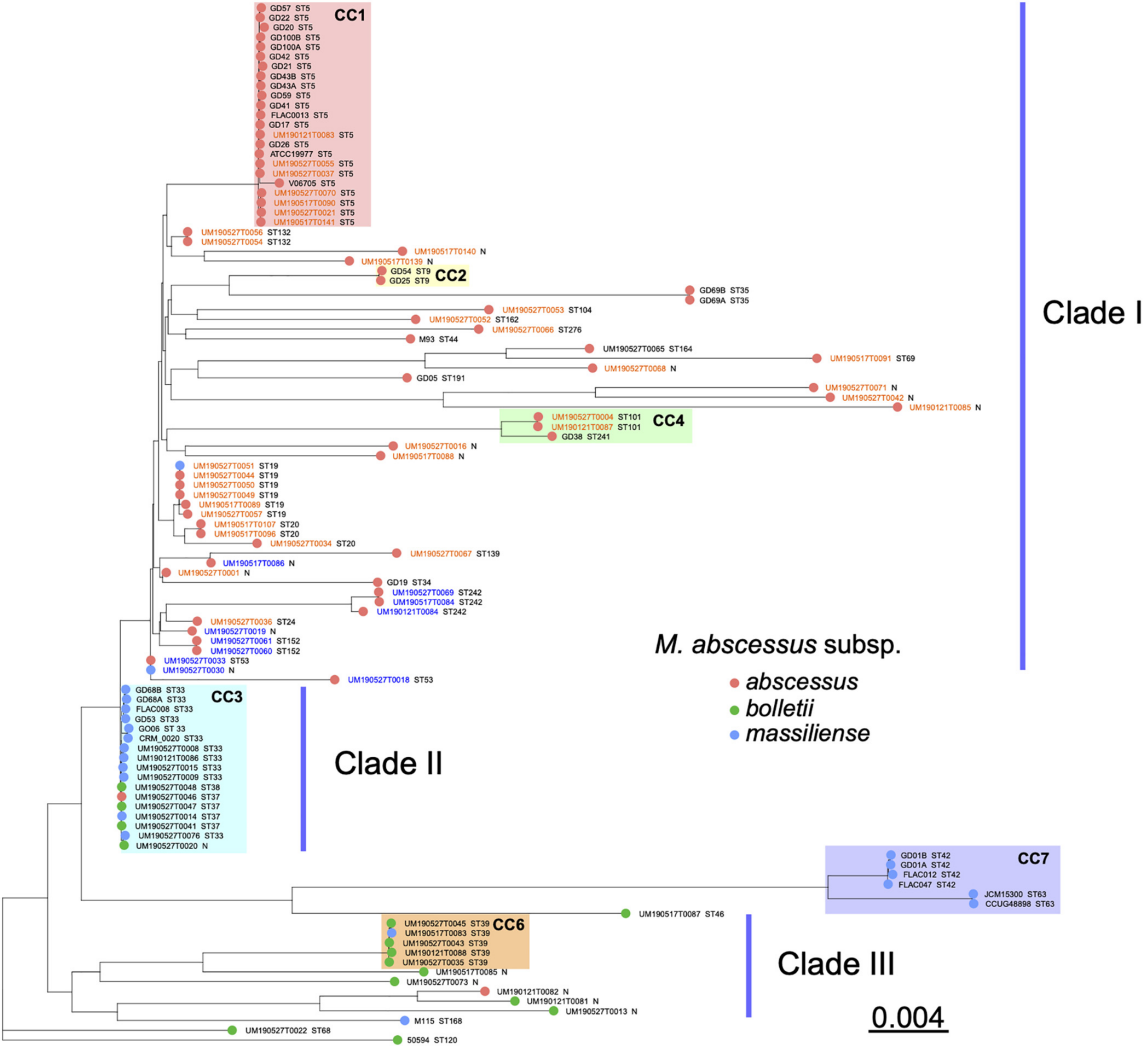
Phylogenetic tree of the 69 MABC clinical isolates and 41 strains with complete genomes in GenBank. M. abscessus strain ATCC 19977 was used as a reference. Strain names in blue or orange indicate those with or without the T28C variant in *erm*(41) gene, respectively, while black indicates those without an intact *erm*(41) gene. Strain names are combined with their sequence types; “N” indicates an unknown sequence type.

The 69 MABC genome sequences (46 M. abscessus, 14 *M. bolletii*, and 9 *M. massiliense*) were combined with another 41 published complete MABC genomes (23 M. abscessus, 4 *M. bolletii*, and 13 *M. massiliense*) to construct a phylogenetic tree (Fig. 2 and Table S2). Most sequenced MABC strains in the present study are distributed into three clades (clades I to III). Six previously reported clonal complexes (CCs) (CC1 to -4, -6, and -7, from the PubMLST profile) were identified. CC1 (23 strains), CC2 (2 strains), and CC4 (3 strains) are formed mostly by M. abscessus ST5, ST9, and ST101 strains, respectively. CC7 is formed by 6 *M. massiliense* strains of ST42 and ST63. CC3 consists of 17 *M. massiliense* strains, most of which belong to ST33 and ST37; interestingly, among these strains, 4 were identified as *M. bolletii*, and 1 was identified as M. abscessus. CC6 involves 5 ST39 strains: 4 are *M. bolletii*, and 1 is M. abscessus. In CC1, 7 M. abscessus clinical isolates show a close relationship (with an average of 667 SNP differences) with published strains originating from France (M. abscessus ATCC 19977 and V06705) and the United States (M. abscessus FLAC013) (Table S9) (30–32). In addition, 7 clinical isolates (UM190121T0086 and UM190527T008, -09, -15, -20, -30, and -76) in CC3 show an average of 471 SNP differences compared to the reference strain *M. massiliense* FLAC008 obtained from the United States (30). In contrast, *M. massiliense* strains FLAC012 (30) and CCUG48898 (33) show a distant relationship compared to the 69 clinical isolates, with approximately 58,891 SNP variations detected (Table S9). Among the 69 strains sequenced in this study, 7, 11, 2, and 5 strains belong to CC1, -3, -4, and -6, respectively, and 46, 11, and 10 are grouped in clades I, II, and III, respectively. It seems that the MABC subspecies identified by a combination of the *hsp65*, *rpoB*, and 16S rRNA genes show a polyphyletic distribution in the constructed phylogenetic tree (Fig. 2). Considering a previous study that revealed inconsistent classifications of MABC strains using *rpoB* (34), we postulate that the current classification of MABC subspecies might be insufficient to distinguish the three subspecies, and a systematic, whole-genome-based analysis is urgently required to improve the taxonomic classifications of MABC strains.

## DISCUSSION

The incidence and prevalence of MABC infections are increasing globally, especially in developed countries with a declining incidence of Mycobacterium tuberculosis infection (35). The Mycobacterium abscessus complex is a major group of nontuberculous mycobacteria and is ubiquitously distributed in various environments. MABC strains are increasingly causing many infections, and pulmonary disease is of the most concern. In Japan and America, rapidly growing mycobacteria cause approximately 5% of pulmonary infections, 80% of which are due to MABC strains (5). MABC has overtaken the Mycobacterium avium complex as the most common nontuberculous mycobacterium causing lung disease in East Asia (36–38). MABC strains have significant genomic diversity, and comparative genomic analyses will assist in a better understanding of transmission, population structure, and critical features such as virulence factors and antimicrobial resistance genes, thereby improving the treatment and control of MABC infections. Previous genomic analyses focused mainly on MABC strains isolated from CF patients (14, 16, 17, 39). However, CF is most common in the Caucasian population but extremely rare in parts of Asia, including China (15, 40). Therefore, there is an urgent need to analyze MABC strains causing non-CF pulmonary infections. In the present study, we have discovered resistance, virulence, and phylogenetic features of MABC strains isolated from Shanghai Pulmonary Hospital between 2014 and 2016. To the best of our knowledge, this is the first large-scale comparative genomic study of MABC strains isolated from patients with non-CF pulmonary infection in China.

An analysis of diagnostic information reveals that 59% (38 of 64) of the patients were initially diagnosed with pulmonary diseases other than nontuberculous mycobacterial infections (see Table S1 in the supplemental material); 5 of these patients had tumors, and 1 MABC infection was discovered while the patient was taking a regular physical examination, indicating that MABC infection can be widespread in the population and sometimes difficult to diagnose. Furthermore, pulmonary shadow and bronchiectasis were characterized in 16 and 28 patients, respectively; they represent the two major clinical manifestations of MABC pulmonary infections, similar to previous reports (41, 42).

Over the past decades, whole-genome sequencing has become a popular technology to identify functional gene contents, including critical genes encoding virulence and antimicrobial resistance. Comparative analyses show significant diversity in MABC genomes, with 27 individual STs identified among 51 strains. These genomes harbor an array of genes conferring resistance to both first-line and secondary antimycobacterials. The wide presence of these genetic determinants and their unique intrinsic mechanisms such as a waxy cell envelope and slow growth together make mycobacteria a nightmare for current antimycobacterial chemotherapies. Interestingly, only 4% (3/69) of the strains harbor genetic mutations in the *rrl* hot spot, including a 2271A>G mutation and a 2823T>C mutation in the *rrl* gene of ATCC 19977; this is consistent with the recent discovery of a low incidence of *rrl* mutations in MABC strains isolated from non-CF patients in Asia (18). In addition, it seems that the T28C variant in the macrolide resistance gene *erm*(41) is present in a small cluster of M. abscessus isolates (Fig. 2), as previously reported (18). Analyses also show that the 69 examined MABC isolates contain a large number (80 to 114) of virulence-associated genes, accounting for approximately 2% of their genomes (Fig. 1).

Since the first complete M. abscessus genome was released in 2009 (31), there have been over 1,000 MABC genomes to date in the GenBank database. The taxonomy of MABC has changed multiple times owing to the significant phenotypic similarities between strains. Recently, MABC strains were subclassified into three subspecies, *M. massiliense*, *M. bolletii*, and M. abscessus, based on DNA hybridization and whole-genome alignment results (14, 39, 43, 44); however, debates on taxonomic classifications still exist (39, 45, 46). In clinical microbiology laboratories, the *hsp65*, *rpoB*, and 16S rRNA genes are used as standard markers to identify the three subspecies. In the present phylogenetic study based on a recombination-free core-genome alignment, it is discovered that several strains of one subspecies are “misplaced” with strains of another subspecies (Fig. 2). For instance, UM190527T0051 and UM190527T0030 were identified as *M. massiliense* using marker genes but clustered with published M. abscessus strains in the phylogenetic tree, whereas strains UM190121T0082 and UM190527T0046 were identified as M. abscessus but clustered with published *M. bolletii* and *M. massiliense* strains. These controversial classifications indicate the insufficiency of current marker genes; hence, a set of novel markers for precise identification based on comparative genomic analysis is greatly needed for both phylogenetic study and precision diagnosis of MABC infections.

### Conclusions.

Overall, this is the first genomic study of MABC clinical isolates from pulmonary patients in China. With comparative analyses, we identified critical genes associated with antimicrobial resistance, virulence, and mobile elements in MABC genomes. We also explored the taxonomic structure of these isolates by constructing a phylogenetic tree. These findings provide a mechanistic understanding of evolution and pathogenesis, which could be valuable for developing novel and effective antimicrobial therapies against these MABC pathogens in China.

## MATERIALS AND METHODS

### Strains and antimicrobial susceptibility testing.

All isolates were cultured in Middlebrook 7H10 broth (BD, France) supplemented with 10% (vol/vol) oleic acid-albumin-dextrose-catalase (Thermo Fisher Scientific, USA). The cultures were incubated at 37°C for 7 days. Only the first MABC isolate for each patient was included (duplicates were excluded).

The Ethics Committee of SPH exempted this study from ethical review because the assessments of the bacteria were part of routine hospital laboratory procedures. All specimens in this study were fully anonymized before they were accessed. No personal patient data are reported in this study, and patient consent was considered to not be required. The 2007 American Thoracic Society-Infectious Diseases Society of America guidelines were employed in our study for the diagnosis of NTM pulmonary disease (5); they include clinical symptoms, chest scan, and microbiological criteria (e.g., laboratory culture).

These isolates and the reference strain M. abscessus ATCC 19977 were subject to culture-based drug susceptibility testing (DST) for the following 15 antimicrobials (Thermo Fisher Scientific) according to the manufacturer’s instructions: amikacin (AMK), ciprofloxacin (CIP), moxifloxacin (MXF), trimethoprim-sulfamethoxazole (SXT), linezolid (LZD), ceftriaxone (47), cefepime (FEP), cefoxitin (FOX), tobramycin (TOB), tigecycline (TGC), minocycline (MIN), doxycycline (DOX), amoxicillin-clavulanic acid (AMC), imipenem (48), and clarithromycin (14). The MICs of the 15 antimicrobials for the 69 MABC isolates were determined using Sensititre RapmycoI MIC plates (Thermo Fisher Scientific) and the broth microdilution method, as recommended in Clinical and Laboratory Standards Institute (CLSI) guideline M24, 3rd ed. (49), and CLSI supplement M62, 1st ed. (50). For those antimicrobials not in the guidelines (AMC, FEP, CRO, MIN, and TGC), previously reported criteria were used for interpretation (51).

### Genome sequencing and assembly.

A bacterial log-phase culture was collected, and genomic DNA was extracted using the SDS method as previously described (52). The harvested DNA was detected by agarose gel electrophoresis and quantified using a Qubit 2.0 fluorometer (Thermo Scientific). A total of 1 μg of DNA per sample was used for library preparation. Specifically, sequencing libraries were generated using the NEBNext Ultra DNA library prep kit for Illumina (New England BioLabs [NEB], USA) according to the manufacturer’s recommendations, and index codes were added to each sample. DNA samples were fragmented by sonication to a size of 350 bp, and DNA fragments were then end polished, poly(A) tailed, and ligated with the full-length adaptor for further PCR amplification and subsequent sequencing using the Illumina NovaSeq PE150 platform (Novogene Bioinformatics Technology Co., Ltd.).

Raw reads were subjected to adaptor trimming and quality filtering (quality score of >20). For each genome, SOAP denovo v2.04 (53), SPAdes v3.10.0 (54), and ABySS v1.3.7 (55) were employed to produce independent assemblies, which were subsequently reconciled by CISA v1.3 (56) and gap-filled by GapCloser v1.12 (53), and small fragments (<500 bp) were filtered out to yield the final assembly. Specifically, for UM190527T0031, -32, -58, -59, -62, and -65 and UM190517T0116 and -117, the reads were first decontaminated using CONSULT (57) with all complete mycobacterial genomes from the GenBank database as a reference. The remaining clean reads were then employed for the following analyses, including genome assembly and annotation.

### Genome annotation.

Genome component prediction included the prediction of coding genes, repetitive sequences, noncoding RNA, genomic islands, transposons, prophages, and clustered regularly interspaced short palindromic repeat (CRISPR) sequences.

The draft assemblies were annotated using Prokka v1.13.3 (58) with default settings. Interspersed repetitive sequences and tandem repeats were predicted using RepeatMasker v4.0.5 (59) and TRF v4.09 (60). Genomic islands, transposons, prophages, and CRISPR sequences were predicted using IslandPath-DIOMB v0.2 (61), transposonPSI (62), PHASTER (63), and CRISPRFinder (64), respectively. Secretory proteins were predicted by SignalP v4.1 (65), and the prediction of type I to VII proteins secreted by pathogenic bacteria was based on EffectiveT3 v1.0.1 (66). VFDB (Virulence Factors of Pathogenic Bacteria Database) v2.0.4 (67) and CARD (Comprehensive Antimicrobial Resistance Database) v2.0.4 (strict algorithm) (68) were used to perform the above-described analyses. BLASTP analysis was conducted with a combination of a coverage of >90% and an identity of >90%. Multiple-locus sequence typing (MLST) was performed using PubMLST (69). Transposons were predicted using BacAnt (70) with an identity of >80% and a coverage of >50%.

### Core-SNP identification and phylogenetic tree construction.

Core-genome SNPs (single nucleotide polymorphisms) were detected by mapping reads to the M. abscessus ATCC 19977 reference genome (GenBank accession number GCA_000069185) using Snippy v4.4.5 (https://github.com/tseemann/snippy). The core SNPs were concatenated and aligned using the snippy-multi script. A maximum likelihood tree was constructed based on the core-SNP alignment using IQ-TREE 2 (using a general time-reversible model with ascertainment bias correction, with 1,000 bootstraps) and then visualized using ggtree v2.4.1 (71, 72).

### Data availability.

The draft genomes of the 69 MABC isolates have been deposited in the GenBank database under the accession number PRJNA832057. The raw reads have been deposited in the SRA (Sequence Read Archive) database with the accession numbers SRX15045449 to SRX15045517. All scripts and data are available from the corresponding authors upon request.
